# Determination and Difference Analysis of Phenolic Compounds in Smokers' Saliva and Mainstream Smoke

**DOI:** 10.1155/2022/6788394

**Published:** 2022-09-29

**Authors:** Xiaoxi Si, Jianyun Yang, Fengmei Zhang, Ruizhi Zhu, Chunbo Liu, Wei Jiang, Qingpeng Shen, Pei He, Shiyun Tang, Zhenjie Li, Zhihua Liu, Junheng You, Zhang Di

**Affiliations:** ^1^Yunnan Key Laboratory of Tobacco Chemistry, R&D Center of China Tobacco Yunnan Industrial Co. Ltd., Kunming 650231, China; ^2^Faculty of Environmental Science & Engineering, Kunming University of Science and Technology, Kunming 650500, China; ^3^School of Resources and Environment, Linyi University, Linyi 276005, China

## Abstract

To study the differences in phenolic compounds between tobacco smokers' saliva and mainstream smoke, a method was developed for the analysis of 12 phenolic compounds in saliva and mainstream smoke based on ultrahigh-performance liquid chromatography with fluorescence detection (UPLC-FLD). The contents and distributions of phenolic compounds in tobacco smokers' saliva and mainstream smoke were compared. The results were as follows: (1) Phenolic compounds were quantitatively analyzed by the internal standard method using 4-fluorophenol as an internal standard. For smokers' saliva samples, the limits of quantification (LOQs) ranged from 2.2 to 19.1 *μ*g/L, and the recoveries were from 80.2% to 119.2% at the three spiked levels. For mainstream smoke samples, the LOQs ranged from 0.03 to 0.26 *μ*g/cig, and the recoveries ranged from 84.9% to 107.0% at the three spiked levels. (2) The contents of phenolic compounds from 14 cigarettes in mainstream smoke and smokers' saliva were determined. In mainstream smoking, the main phenolic compounds were hydroquinone, catechol, phenol, *meta*- and *para*-Cresol, and *o*-methylhydroquinone. In smokers' saliva, the main phenolic compounds were phenol and *meta*- and *para*-Cresol and the contents of phenolic compounds in smokers' saliva from different cigarettes were significantly different. (3) The content distribution patterns of phenolic compounds in smokers' saliva differed from those in mainstream smoke. The predominant phenolic compound in mainstream smoke was dihydroxybenzene, while monophenols predominated in smokers' saliva. (4) The contents of phenolic compounds from five kinds of cigarettes were analyzed in the saliva of different smokers using principal component analysis, which indicated that cigarettes with different sensory effects were clearly distinguished by differences in the contents of phenolic compounds in saliva.

## 1. Introduction

Phenolic compounds are among the main components of cigarette smoke and are highly related to the safety of cigarettes and the sensory quality of smoke [1]. There are two roles for phenolic compounds in smoke. On the one hand, phenol, methylphenol, hydroquinone, and other simple phenolic compounds give a pungent smell, which is associated with intense irritation and corrosion of the eyes, mucosa, skin, and so on [2]. These components are known to be harmful in cigarette smoke [3]. On the other hand, some phenolic compounds have special odors that provide excellent smoke properties and are not suppressed by other fragrance agents [1, 4]. For example, eugenol, 2-methoxy-4-methylphenol, has a strong fragrance and has been used as an important ingredient [5].

There are large amounts of phenolic compounds in mainstream smoke, and some studies have reported that there is a connection between phenolic compounds and sensory quality [6]. Nevertheless, reports on the mechanisms of detection of phenolic compounds by the human senses are scarce. Cheng et al. reported that the amounts of 7 phenolic compounds, dihydroxybenzene, hydroxyphenol, *o*-dihydroxybenzene, phenol, *m*-Cresol, *p*-Cresol, and *o*-Cresol, were significantly correlated with some sensory indicators, including the physiological strength, concentration, irritancy, and humidification degree of smoke, but had little influence on the fragrance, offensive odor, and aftertaste of smoke [7]. In another study, Yu et al. [8] applied a special filter to adsorb phenolic compounds, which resulted in an obvious change in sensory quality. Basically, phenolic compounds in mainstream smoke are dissolved and adsorbed by smokers' saliva and then have physiological effects on the human body, including sensory effects [9]. However, it is not known how phenolic compounds dissolve into saliva from smoke, and then, act on the human body. Therefore, further research on the phenolic compounds in smokers' saliva would help explain the effects of phenolic compounds on the human body.

There have been many studies on phenolic compounds in cigarette smoke [10, 11]. However, few studies have analyzed phenolic compounds in saliva. In addition, pretreatment technologies for phenolic compounds include liquid‒liquid extraction, column chromatography, solid-phase extraction, and derivatization. These pretreatment processes are quite complicated, and sample loss or oxidation frequently occurs [11, 12]. The common methods for the determination of phenolic compounds are gas chromatography–mass spectrometry (GC–MS) [13, 14] and high-performance liquid chromatography (HPLC) [15, 16]. When GC‒MS methods are applied, derivatization is usually necessary since phenolic compounds are oxidized or decomposed at high temperatures [11, 14]; furthermore, derivatization must be performed under anhydrous conditions, which is extremely complex, and the results may not be as accurate as required. In contrast, HPLC is performed at room temperature and is usually carried out with a diode array detector [15] or fluorescence detector [16]; fluorescence detectors exhibit high selectivity and are resistant to matrix interference [17, 18].

In summary, studies on phenolic compounds in smokers' saliva and mainstream smoke, including their contents and distributions, are of great significance in further evaluating the effects of phenolic compounds on the sensory quality and safety of cigarette smoke.

## 2. Materials and Methods

### 2.1. Chemicals, Reagents, and Calibration Solutions

Fourteen kinds of flue-cured cigarettes were purchased in the Chinese market. Acetonitrile (HPLC grade) was purchased from Merck (Darmstadt, Germany). Acetic acid (analytical grade) was purchased from Xilong chemical group (Guangdong, China). Hydroquinone, hydroxyphenol (also known as resorcinol), *o*-methylhydroquinone, *o*-dihydroxybenzene (also known as catechol), phenol, *m*-Cresol, *p*-Cresol, *o*-Cresol, 2-methoxy-4-methylphenol, 2,6-dimethylphenol, 2-ethylphenol (also known as phlorol), 1-allyl-3-methoxy-4-hydroxybenzene (also known as eugenol), and 4-fluorophenol (purities > 98%) were obtained from Dr. Ehrenstorfer (Augsburg, Germany).

The phenolic standards were dissolved in methanol at a concentration of 1000 mg/L to prepare primary stock standard solutions. The internal standard, 4-fluorophenol, was dissolved in methanol at a concentration of 100 mg/L to prepare stock internal standard solutions. The primary stock standard solutions were further diluted with deionized water to make secondary stock standard solutions of 50 mg/L for each target phenolic compound.

The calibration solutions used for the quantification of phenolic compounds in mainstream smoke were made from dilutions of the secondary stock standard solutions with 1% acetic acid solution. Then, 0.005 mL, 0.02 mL, 0.1 mL, 0.4 mL, and 1.0 mL of the secondary stock standard solutions were accurately transferred into a 10 mL volumetric flask, and 50 *μ*L of stock internal standard solution was added. Finally, the solution was diluted to 10 mL with 1% acetic acid aqueous solution.

The calibration solutions used for the detection of phenolic compounds in saliva were made from dilutions of the secondary stock standard solutions with 1% acetic acid solution. Secondary stock standard solutions (0.005 mL, 0.02 mL, 0.1 mL, 0.4 mL, and 1.0 mL) were accurately transferred into a 10 mL volumetric flask and diluted to 10 mL with water, and then, 1.0 mL of each solution was added to 15 *μ*L of acetic acid and 20 *μ*L of stock internal standard solution.

### 2.2. Instrumentation

An Acquity™ ultra performance liquid chromatograph with a fluorescence detector was obtained from waters (Milford, MA, USA). An ultrasonic cleaner with a power ≥200 W was purchased from Kunshan Ultrasonic Instrument Co., Ltd. (Kunshan, China). A BT224S electronic balance with 0.0001 g capacity was purchased from Sartorius (Göttingen, Germany). A VX-200 Vortex Mixer was obtained from Labnet (NJ, USA). A Milli-Q Water Purification System was purchased from Millipore (Billerica, USA). Salivette tubes were obtained from Sarstedt (Nuembrecht, Germany). Membrane filters (0.22 *μ*m) were obtained from Tianjin Jinteng Experimental Equipment Co., Ltd. (Tianjin, China).

### 2.3. Smoke Collection and Pretreatment

The mainstream smoke collection was performed according to YC/T 255-2008 [19]. The total particulate matter of mainstream smoke from 4 cigarettes was collected on a glass fiber filter pad (Cambridge filter pad, CFP). The pad was, then, put into a 200 mL conical flask, and 50 mL of 1% acetic acid aqueous solution (containing 0.5 mg/L 4-fluorophenol) was added accurately. Ultrasonic extraction, then, proceeded for 20 min at room temperature; after resting for 5 min, the extract was filtered through a 0.22 *μ*m microfiltration membrane and analyzed by UPLC-FLD.

### 2.4. Saliva Sample Collection and Pretreatment

Volunteer smokers (*N* = 6) were strictly trained on the saliva collection process, in which the subjects rinsed 2 times with water, put cotton sticks from the saliva tube into the mouth, and chewed 1 time per second for 2 min; these cotton sticks were collected as blank samples. After that, all smokers finished 1 cigarette with 6 fixed puffs, put cotton sticks into their mouths, and chewed 1 time per second for 2 min; these cotton sticks were collected as smoker samples.

The collected cotton sticks were centrifuged at 5000 r/min for 15 min, and the saliva samples were obtained. The saliva samples (1 mL) were placed into a 2.0 mL sample tube, 15 *μ*L of acetic acid and 20 *μ*L of stock internal standard solution were added, and the mixture was agitated at 2000 rpm via a vortex mixer for 2 min. The mixture was, then, filtered through 0.22 *μ*m microfiltration membranes and analyzed by UPLC-FLD.

### 2.5. UPLC-FLD Analytical Conditions

Chromatographic separation was achieved by using a C18 reversed-phase column (100 mm × 2.1 mm i.d. × 1.7 *μ*m·dp) from Waters. An acetic acid (1%) solution was used as mobile phase A, and an acetic acid-acetonitrile-water mixed solution with a 1 : 30 : 69 volume ratio was used as mobile phase B. Gradient elution was performed with 10% B∼100% B from 0∼10 min and 100% B∼100% B from 10∼20 min. The injection volume was 2 *μ*L, a flow rate of 1.0 mL/min was applied, and the temperature was set at 50°C. Samples were analyzed with a fluorescence detector; the wavelengths used for fluorescence detection are shown in Table 1.

## 3. Results and Discussion

### 3.1. Optimization of the Pretreatment Method

Ultrasonic-assisted extraction (UAE) was chosen to extract the phenolic compounds from mainstream cigarette smoke according to YC/T 255-2008 [19]. UAE has proven to be an efficient technique for the extraction of phenolic compounds, as it reduces energy consumption, minimizes extraction times and temperatures, and allows the substitution of organic solvents with nontoxic solvents, such as the aqueous acid solution used in this experiment, which is useful for the extraction of thermolabile compounds [20, 21]. Considering the complexity of smoke matrices, including differences in pH value, studies on acidification and dilution treatment of saliva samples were performed. On the one hand, acidification provides free phenolic compounds and enhances their fluorescence response, resulting in better determination performance [22, 23]; on the other hand, adding organic solvents as diluents and precipitators will reduce the impacts of interfering substances on chromatographic separation as much as possible [24–26].

**Figure 1:**
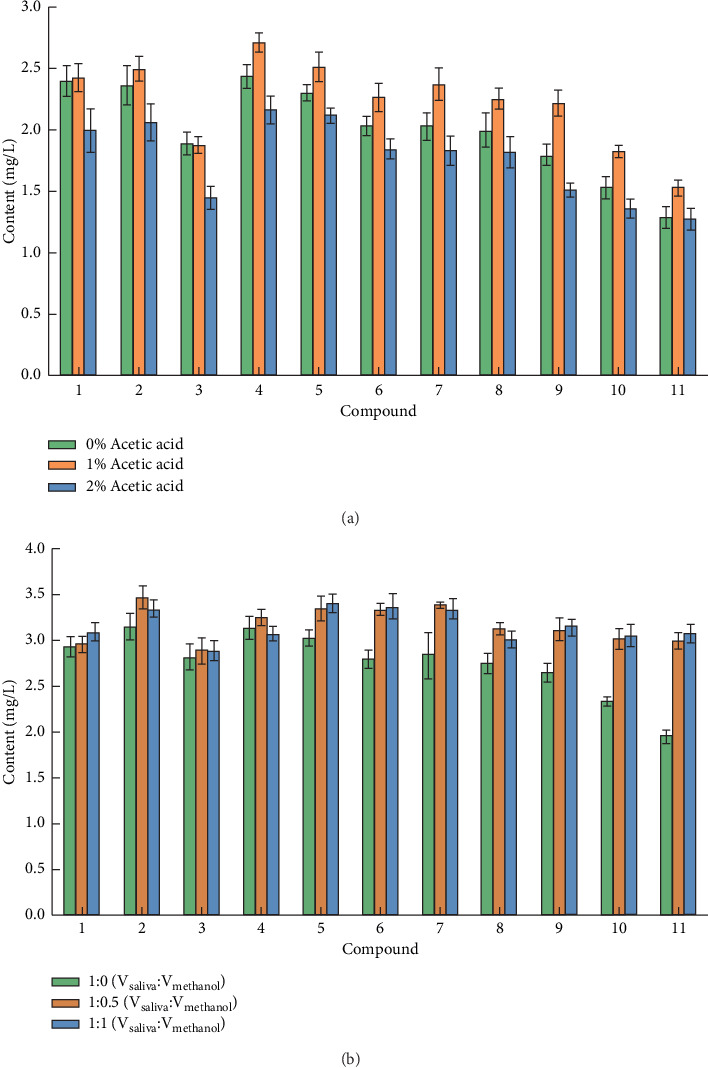
Influence of acidity (a) and proportion of diluent (b) on the determination results for phenolic compounds in saliva samples (*n* = 3). The numbers of compounds are the same as in Table 1.

Since, phenolic compounds exist in different forms under acidic and alkaline conditions, changes in pH and other conditions may affect the fluorescent spectral profile and fluorescence intensity of phenolic compounds. For example, C_6_H_5_OH and C_6_H_5_OH_2_^+^ are the main forms of phenol in acid solutions, but C_6_H_5_O^−^ is the main form in alkali solution. The electronic transition of C_6_H_5_OH_2_^+^ and C_6_H_5_O^−^ is forbidden, and there is no fluorescence [22]. Therefore, certain acidic environmental conditions need to be provided and controlled to enhance and obtain stable fluorescence response values. In this study, acetic acid was used to acidify the saliva samples, and different concentrations of acetic acid, 0%, 1%, and 2%, were tested to identify their influence on the chromatographic separation and fluorescence intensity of the targets. The results showed that 12 phenolic compounds presented similar chromatographic peaks with different concentrations of acetic acid. As shown in Figure 1, the target responses reached a maximum value when the concentration of acetic acid was 1% but were slightly reduced in a 2% acetic acid solution, probably due to the increase in H^+^ concentration, which increased the ionic state of phenolic compounds and weakened the fluorescence intensity [23]. In addition, the sample solvent had acidity consistent with that of the mobile phase and contained 1% acetic acid; thus, the chromatographic separation and response values of the targets were not affected by differences in acidity between the sample solvent and the mobile phase. In summary, 1% acetic acid was used in the experiments.

Saliva contains proteins and many exogenous or endogenous small molecules, which may interfere with the chromatographic separation of phenolic compounds, thus, affecting the detection results. One common method to eliminate matrix effects on analytes is to add organic solvents that are miscible with saliva to precipitate proteins and dilute the sample matrix. Methanol, a protein precipitant commonly used in the pretreatment of biological samples [25, 26], also has good solubility for phenolic compounds. Therefore, methanol was chosen as a diluent, and different dilution ratios, 1 : 0 (V_saliva_ : V_methanol_, undiluted), 1 : 0.5 (V_saliva_ : V_methanol_), and 1 : 1 (V_saliva_ : V_methanol_), were tested. The results showed that there was little difference in chromatographic separation effects between diluted and undiluted samples, and the chromatographic peaks were all sharp and symmetrical. One possible factor was the high selectivity and lack of matrix interference of the fluorescence detector.  Figure 1(b) shows the influence of the diluent ratio on phenolic compound determination in saliva samples. The artificial saliva was mixed with methanol in ratios of 1 : 0, 1 : 0.5, and 1 : 1 to prepare 12 phenolic compound solutions of the same concentration, which were, then, determined after centrifugation. The maximum responses were obtained at artificial saliva-to-methanol ratios of 1 : 0.5 and 1 : 1, which indicated a slight increase in the target fluorescence response when methanol was added. Nevertheless, in consideration of the weaker response enhancement observed when methanol was used as the diluent and since the resulting sample would be too dilute to determine, the saliva samples were pretreated without dilution.

### 3.2. Optimization of Determination Conditions

The best excitation and emission wavelengths for the 12 phenolic compounds and the internal standard 4-fluorophenol were determined by spectral scanning with standard solutions, the results of which are shown in Table 1. The targets were divided into three groups based on the measured optimum excitation and emission wavelengths, and three channels were applied for testing: (1) an excitation wavelength of 295 nm and an emission wavelength of 325 nm; (2) an excitation wavelength of 272 nm and an emission wavelength of 300 nm; and (3) an excitation wavelength of 275 nm and an emission wavelength of 310 nm.

**Figure 2:**
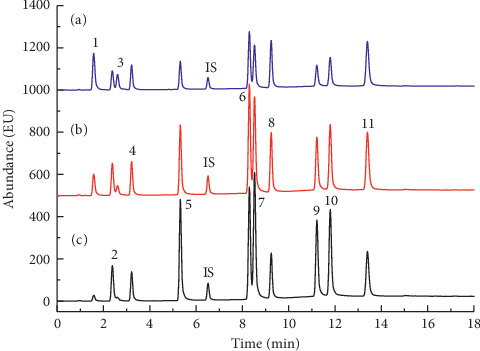
Chromatograms of the standard solution from ultrahigh-performance liquid chromatography with fluorescence detection. The numbers of compounds are the same as in Table 1. (a) Chromatogram detected at 325 nm; (b) chromatogram detected at 310 nm; and (c) chromatogram detected at 300 nm.

The gradient elution conditions and the mobile phase were also optimized, and a 1% acetic acid addition was used to quickly elute and separate the target analytes. Chromatograms of the standard solution under optimized chromatographic conditions are shown in Figure 2. All chromatographic peaks for the target compounds were sharp and symmetrical, indicating good separation. Among the targets, *m*-Cresol and *p*-Cresol were combined in this study since they simultaneously eluted [21]. Compared with YC/T 255-2008 [21], the running time was decreased from 40 min to 18 min, and the number of targets was increased from 7 to 12, which indicated a better method with a higher efficiency.

In addition, the internal standard method was applied for quantitative analysis to improve the accuracy of quantification. 4-Fluorophenol was chosen as the internal standard based on the properties and retention times of the targets. As seen from the figure, 2,4-fluorophenol presented a high response at 3 different detection wavelengths and was well separated from the 12 targets, which means that it was a suitable internal standard for the detection method.

### 3.3. Method Evaluation

Twelve phenolic compounds showed good linearity with correlation coefficients (*R*^*2*^) greater than 0.99. The relative standard deviations (RSDs), limits of detection (LODs), limits of quantification (LOQs), and recoveries are shown in Table 2. For smokers' saliva samples, the LOQs ranged from 2.2 to 19.1 *μ*g/L, and the recoveries were from 80.2% to 119.2% at the three spiked levels of 0.05, 0.5, and 2.0 mg/L. For mainstream smoke samples, the LOQs ranged from 0.03 to 0.26 *μ*g/cig, and the recoveries ranged from 84.9% to 107.0% at spiked levels of 1.0, 5.0, and 25.0 *μ*g/cig. All samples presented reasonable results with good precision, relatively low LODs, and high recovery rates, indicating that the method applied here is reliable and suitable for the determination of phenolic compounds in saliva and mainstream smoke samples.

### 3.4. Content Analyses of Phenolic Compounds in Smokers' Saliva and Mainstream Smoke

**Figure 3:**
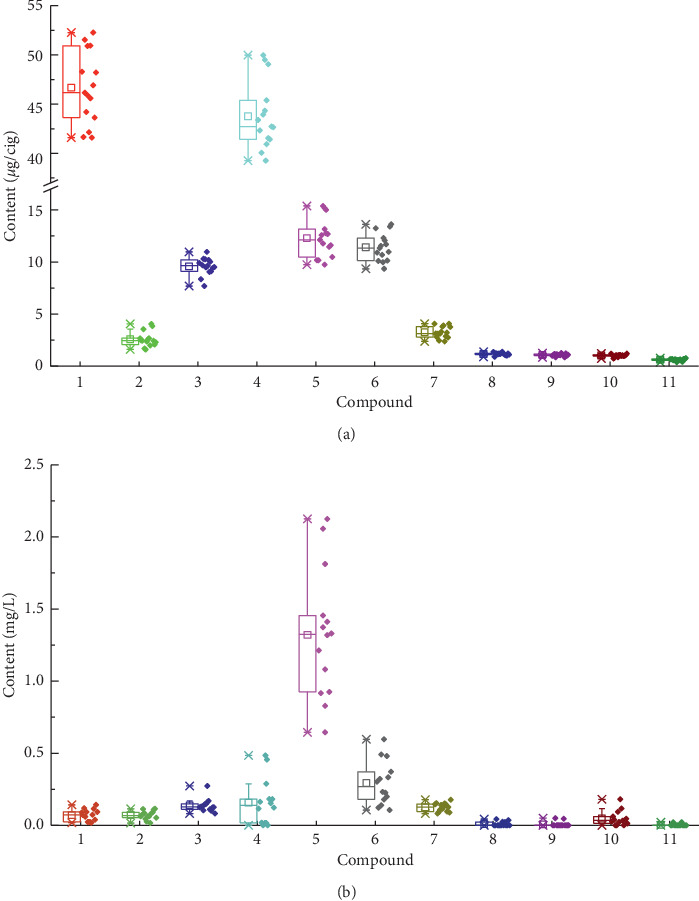
Box plots of phenolic compounds contents in mainstream smoke (a) and smokers' saliva (b) for different cigarettes. The numbers of compounds are the same as in Table 1.

The phenolic compounds from 14 different kinds of cigarettes were investigated in mainstream smoke and smokers' saliva with the method described above; the results are shown in Figure 3. No phenolic compounds were detected in the blank saliva samples of the smokers, which are not shown in the figure. The box plots in Figure 3(a) show that catechol and hydroquinone were the main phenolic compounds in mainstream smoke. In addition, the amounts of phenol, (*m*, *p*)-Cresol, *o*-methylhydroquinone, *o*-Cresol, and resorcinol were relatively high. Low levels of 2-methoxy-4-methylphenol, 2,6-dimethylphenol, phlorol, and eugenol were also detected. The same phenolic compounds were reported in the literature [27]. In addition, the amounts of the same phenolic compounds in mainstream smoke from different cigarettes presented relatively small differences.

The box plots in Figure 3(b) show that phenol and (*m*, *p*)-Cresol were the main phenolic compounds detected in smokers' saliva. In addition, the amounts of catechol, *o*-Cresol, and *o*-methylhydroquinone were relatively high. Lower amounts of hydroquinone, resorcinol, and phlorol were also detected. It is worth mentioning that 2-methoxy-4-methylphenol, 2,6-dimethylphenol, and eugenol were detected in trace amounts below the quantitative limit. In contrast with the phenolic compounds in mainstream smoke, the amounts of the same phenolic compounds from different cigarettes were very different in smokers' saliva.

### 3.5. Variance Analyses of Phenolic Compounds in Smokers' Saliva and Mainstream Smoke

**Figure 4:**
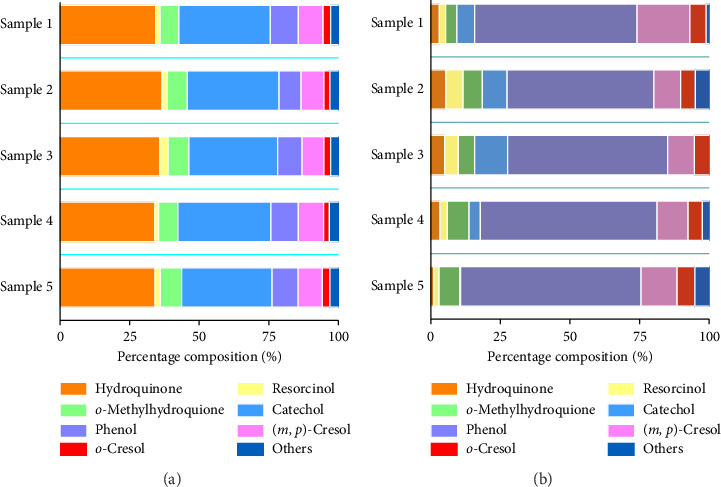
Percentage composition of phenolic compounds in mainstream smoke (a) and in saliva (b) of different tobacco smokers.

Five kinds of cigarettes with the same tar level were chosen for comparison of the distributions of phenolic compounds in mainstream smoke and smokers' saliva. As shown in Figure 4, there were large differences between the distributions of phenolic compounds in mainstream smoke and saliva; dihydroxybenzene was the main phenolic compound in mainstream smoke, but monophenols, including phenol and (*m*, *p*)-Cresol, were the main phenolic compounds in smokers' saliva. There were large differences in the behavior of dihydroxybenzenes (including hydroquinone and catechol) and monophenols (including phenol and (*m*, *p*)-Cresol) during inhalation into the human mouth. Phenolic compounds such as dihydroxybenzene that are present in large amounts in mainstream smoke may be metabolically converted while dissolved in smokers' saliva [28]. Furthermore, because of differences in the harmfulness [29] and odor [30] characteristics of phenolic compounds and because reactions in saliva can transform these compounds, evaluating the variations in the content of phenolic compounds in smokers' saliva would help improve the understanding of phenolic compounds in mainstream smoke and their effects on health and sensory quality.

### 3.6. Phenolic Compounds in Saliva by Principal Component Analysis

**Figure 5:**
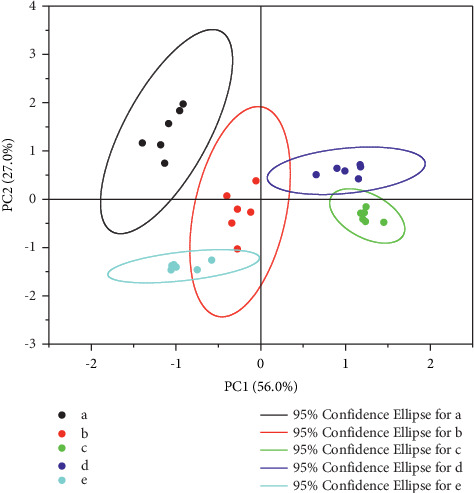
Phenolic compounds contents in smokers' saliva for five kinds of cigarettes (a, b, c, d, and e) determined by principal component analysis.

The above results show that the distribution of phenolic compounds in mainstream cigarette smoke changes significantly after uptake into human saliva through cigarette smoking and that the content of phenolic compounds in the saliva of different cigarette smokers also varies significantly. The type and content of phenolic compounds absorbed and dissolved in saliva directly influence sensory effects and other biological effects in the human body. To study the relationship between phenolic substances in smokers' saliva and the sensory effects of cigarettes, five kinds of cigarettes with the same amount of tar and significant differences in irritation and aroma by sensory evaluation were selected for the experiment. Each kind of cigarette was used by 6 smokers, and then their saliva was collected and determined by the method described above. The contents of phenolic compounds were determined and processed by principal component analysis (PCA), as shown in Figure 5. The contents of phenolic compounds in the saliva of smokers distinguished cigarettes with different sensory effects at the 95% confidence level, except that cigarette B and cigarette E overlapped slightly, probably because there were some similarities in the formulations of cigarettes B and E. Further investigation of the exponential distributions of principal components indicated that among the five types of cigarettes, there were certain differences among the contents of resorcinol, *o*-methylhydroquinone, phenol, and (*m*, *p*)-Cresol in smokers' saliva, and it is suggested that these substances may be the key phenolic substances for distinguishing cigarettes with different sensory effects.

## 4. Conclusions

Phenolic compounds in mainstream smoke and smokers' saliva were quantitatively analyzed by using 4-fluorophenol as an internal standard. The contents of phenolic compounds from 14 cigarettes were determined in mainstream smoke and in smokers' saliva. In mainstream smoke, the main phenolic compounds were hydroquinone, catechol, phenol, *meta*- and *para*-Cresol and *o*-methylhydroquinone. In smokers' saliva, the main phenolic compounds were phenol and *meta*- and *para*-Cresol, and the contents of phenolic compounds in smokers' saliva were significantly different for different cigarettes. The content distribution pattern of phenolic compounds in smokers' saliva differed from that in mainstream smoke. Dihydroxybenzene constituted the largest proportion in mainstream smoke, and monophenols predominated in smokers' saliva. The contents of phenolic compounds from five kinds of cigarettes were analyzed in the saliva of different smokers by using principal component analysis, and the results indicated that cigarettes with different sensory effects could be clearly distinguished by differences in the contents of phenolic compounds in saliva.

## Figures and Tables

**Table 1:** Wavelengths for fluorescence detection.

No.	Compound	Maximum excitation wavelength (nm)	Maximum emission wavelength (nm)	Selected excitation wavelength (nm)	Selected emission wavelength (nm)	Retentiontime (min)
1	Hydroquinone	295	325	295	325	1.60
2	Resorcinol	273	302	272	300	2.41
3	*o*-methylhydroquinone	295	325	295	325	2.65
4	Catechol	274	310	275	310	3.25
5	Phenol	271	297	272	300	5.38
6	(*m, p*)-Cresol	276	305	275	310	8.36
7	*o*-Cresol	272	298	272	300	8.59
8	2-methoxy-4-methylphenol	278	312	275	310	9.32
9	2,6-dimethylphenol	270	298	272	300	11.37
10	Phlorol	272	299	272	300	11.89
11	Eugenol	278	310	275	310	13.68

**Table 2:** LODs, LOQs, RSDs, and recoveries for 12 analytes in saliva and mainstream smoke samples.

Compound	Saliva				Mainstream smoke			
	RSD/% (*n* = 7)	LOD/(*μ*g/L)	LOQ/(*μ*g/L)	Recovery/% (*n* = 3)	RSD/% (*n* = 7)	LOD/(*μ*g/cig)	LOQ/(*μ*g/cig)	Recovery/%(*n* = 3)
Hydroquinone	1.9	3.0	10.0	110.7–119.0	2.8	0.05	0.16	97.2–112.01
Resorcinol	3.3	1.7	5.6	112.5–119.2	3.4	0.02	0.08	95.5–107.1
*o*-methylhydroquinone	1.6	5.8	19.1	85.1–100.9	2.1	0.08	0.26	88.1–103.2
Catechol	0.5	1.7	5.7	111.5–119.1	1.4	0.03	0.09	96.4–114.6
Phenol	2.6	0.7	2.2	104.7–119.0	2.9	0.01	0.04	93.8–117.0
(*m, p*)-Cresol	0.2	0.6	1.9	96.6–117.2	1.5	0.01	0.03	92.1–114.1
*o*-Cresol	1.3	0.7	2.2	98.2–115.4	1.8	0.01	0.04	94.2–107.4
2-methoxy-4-methylphenol	2.6	1.0	3.4	90.7–118.5	3.4	0.02	0.06	93.7–115.5
2,6-dimethylphenol	2.0	1.1	3.5	84.4–109.7	2.2	0.02	0.06	86.4–107.7
Phlorol	1.3	0.8	2.7	81.3–93.3	2.0	0.01	0.04	88.4–102.1
Eugenol	1.8	0.9	2.8	80.2–90.4	2.8	0.01	0.04	84.9–98.8

## Data Availability

The data used to support the findings of this study are included within the article.
